# Australasian Recurrent Pregnancy Loss Clinical Management Guideline 2024 Part I

**DOI:** 10.1111/ajo.13821

**Published:** 2024-06-27

**Authors:** Adriana Suker, Ying Li, Danielle Robson, Anthony Marren, the Australasian CREI (Certificate of Reproductive Endocrinology and Infertility) Consensus Expert Panel on Trial Evidence (ACCEPT) group, Rob Norman, Gab Kovacs, Lucy Prentice, Elizabeth Glanville, Leigh Searle, Olivia Stuart, Vicki Nisenblat, Neil Johnson, Yousif Alyousif, Maree Lee, Kate Burston, Michael Chapman, Phill McChessney, Clare Boothroyd, Neerja Kamal, Anne Clark, Violet Kieu, Rituparna Dutta, Lynn Burmeister, Ashleigh Smith, Louise Hull, Vamsee Thalluri, Giselle Crawford, Beverley Vollenhoven, Sarah Hunt, Nicole Hope, Sameer Jakar, Jinny Foo, Iris Wang, Rabia Shaik, Seema Mohiuddin, Fleur Cattrall, Peter Leung, Roger Hart, Alison Gee, Katrina Rowan, Michele Kwik, Tamara Hunter, Nalini Gayer, Raelia Lew, Rebecca Mackenzie‐Proctor, Anusch Yazdani, Rob Lahoud, Cheryl Phua, Shannon Zawada, Gabrielle Dezarnaulds, Sonal Karia, Devini Ameratunga, Vanessa Ross, Manny Mangat, Raewyn Tierney, Shadi Khashaba, David Greening, Janelle McDonald, Myvanwy McAveen, Danielle Robson, Sebastian Leathersich

**Affiliations:** ^1^ Department of Obstetrics & Gynaecology Liverpool Hospital Sydney New South Wales Australia; ^2^ Department of Reproductive Endocrinology & Infertility Royal Prince Alfred Hospital, Women & Babies Sydney New South Wales Australia

**Keywords:** recurrent pregnancy loss, recurrent miscarriage, guideline, RPL

## Abstract

Guidelines for the investigation and management of recurrent pregnancy loss (RPL) have been developed in Europe, USA and UK, but there is currently no Australasian guideline. The Australasian Certificate of Reproductive Endocrinology and Infertility Consensus Expert Panel on Trial Evidence group has prepared a two‐part guideline to provide guidance on the management of RPL. In Part I chromosomal, anatomical, and endocrine factors are outlined along with relevant recommendations for clinical management, levels of evidence and grades of consensus. In Part II thrombophilia, autoimmune factors, infective, inflammatory, and endometrial causes, environmental and lifestyle factors, male factor and unexplained causes will be outlined.

## INTRODUCTION

Pregnancy loss is defined as the spontaneous loss of pregnancy before the fetus reaches viability.^1^ It has been estimated that between 12% to 15% of clinically recognised pregnancies result in spontaneous loss.^2^ However, the rate of subclinical pregnancy loss is much greater.^2^, ^3^

The most recent international ‘consensus’ on the definition of recurrent pregnancy loss (RPL) is two or more pregnancy losses prior to 24 weeks gestation with the same partner.^4^, ^5^ Due to the changing gestation of viability, the Australasian CREI (Certificate of Reproductive Endocrinology and Infertility) Consensus Expert Panel on Trial Evidence (ACCEPT) group has chosen to change this definition to prior to 20 weeks gestation. Approximately 5% of women will experience two pregnancy losses (ASRM 2012)^4^ and, albeit low quality evidence, the diagnostic yield appears to be the same whether evaluating women with two or more pregnancy losses.^6^ Maternal age and number of previous pregnancy losses independently predict future pregnancy losses (Table 1).

**Table 1:** Rate of pregnancy loss with maternal age

Age (years)	Pregnancy loss (%)^†^
20–24	11
25–29	12
20–24	15
35–39	25
40–44	51
>45	93

^†^ Data extracted from Li and Marren.^88^

The aetiology of RPL can be divided into the following groups: chromosomal, anatomical, thrombophilia, endocrine, autoimmune, infective and inflammatory endometrial, environmental, male factors, and unexplained.

Guidelines have been developed for the investigation and management of RPL by the European Society of Human Reproduction (ESHRE),^5^ American Society for Reproductive Medicine (ASRM),^4^ and the National Institute for Health and Care Excellence accredited Green‐top Guidelines for the United Kingdom.^1^ However, there is currently no Australasian guideline for RPL.

In an effort to provide guidance to clinicians working with infertile couples, this document, produced by the ACCEPT group, provides an Australasian consensus statement on the current management of RPL in infertile couples.

## MATERIALS AND METHODS

MEDLINE, EMBASE, PubMed, and the Cochrane Database of Systematic Reviews were searched using the terms ‘recurrent’, ‘pregnancy loss’, ‘miscarriage’, ‘spontaneous abortion’, ‘recurrent abortion’ in addition to terms pertaining to aetiology (see Appendix S1). Studies were limited to humans and English language. The date of the last search was March 2023.

This document uses the Australian National Health and Medical Research Council (NHMRC) levels of evidence as outlined in Table 2.^7^ A comprehensive literature review was completed by four authors, then disseminated to content experts, prior to being reviewed by the ACCEPT group. The ACCEPT group consists of the Australian and New Zealand Society of Reproductive Endocrinology and Infertility (ANZSREI) the governance structure of which can be viewed on the group's website (anzsrei.com). The evidence was reviewed and presented to the ACCEPT group in 2023 and classified according to the nomenclature listed in Table 3 to define levels of agreement regarding the statements within this document. Consensus statements were modified as required. All contributing ACCEPT group clinicians in attendance are listed in the Acknowledgements. Recommendations for overall definition and management of RPL are given in Table 4.

**Table 2:** Levels of evidence

Level of evidence	Intervention
GPP Level I Level II Level III‐1 Level III‐2 Level III‐3 Level IV	Good practice principle Systematic review of Level II studies Randomised controlled trial Pseudorandomised controlled trial A comparative study with concurrent controls A comparative study without concurrent controls Case series with either post‐test or pre‐test/post‐test outcomes

**Table 3:** Agreement consensus

Consensus	Symbol
Unanimous Unanimous with caveat Majority No consensus	*α* *β* *γ* *δ*

**Table 4:** Recommendations for overall definition and management of recurrent pregnancy loss (RPL)

Statement	Level of evidence
	Grade of consensus
RPL is defined by at least two clinical pregnancy losses prior to 20 weeks gestation	GPP Consensus grade β
Women with RPL should be managed by a medical practitioner with the necessary skill set, expertise, and where available multi‐disciplinary support	GPP Consensus grade α

## RESULTS

### Chromosomal factors

#### Embryonic chromosomes

Aneuploidy (mainly trisomy) is the most common cause of first trimester loss^8^, ^9^, ^10^ in both sporadic and RPL and is reported in up to 67% of cases.^11^, ^12^ There is a relationship between advancing maternal age and the rate of aneuploidy in embryos.^13^

#### Parental chromosomal rearrangement

Balanced translocations and/or inversions are found in approximately 4.7–14.6% of couples who have two or more miscarriages.^14^, ^15^ Carriers of balanced translocations and inversions have a normal phenotype but have a higher propensity to create chromosomally unbalanced embryos, which may fail to implant or result in later pregnancy loss, in up to 60–80% of embryos.^14^ Parental karyotype should be requested in RPL where there is an unbalanced structural chromosome rearrangement on analysis of products of conception (POC). Parental karyotypes should also be considered in other couples after individual risk assessment, including factors such as maternal age and family history of recurrent miscarriage in sibling or parent,^16^ and should be requested prior to commencing additional treatment options such as *in vitro* fertilisation (IVF).

##### Management

Chromosomal aneuploidy is the most common cause of pregnancy loss, and molecular chromosome analysis alone provides an answer to couples in about two‐thirds of cases.^12^ Trisomies represent 60% of all cytogenetic abnormalities, monosomy X is reported in about 20% and triploidy occurs in about 15% of cases.^17^ Analysis of POC tissue via a molecular‐based approach (array‐comparative genomic hybridisation or single nucleotide polymorphism array) should be offered in the RPL setting. A molecular‐based approach is preferred due to higher tissue culture failure rates and maternal cell contamination rates in karyotype analysis of POC.^5^, ^12^ Table 5 outlines the advantages and disadvantages of the various techniques.

**Table 5:** Summary of advantages and disadvantages of chromosome testing techniques

	Advantages	Disadvantages
Array‐comparative genomic hybridisation (CGH)	Does not require live cells, allowing for retrospective analysis Can detect microdeletions/duplications Abnormalities detected throughout the entire genome Reduced maternal cell contamination effect	Unable to detect balanced chromosome rearrangements (translocations, inversions) Unable to detect triploidy, tetraploidy, low‐level mosaicism Unable to detect maternal cell contamination
Single nucleotide polymorphism array	Does not require live cells, allowing for retrospective analysis Can detect microdeletions/duplications Abnormalities detected throughout the entire genome Can detect maternal cell contamination Can provide information about parental origin of aneuploidy Can detect some placental mosaicism Can detect triploidy	Unable to detect balanced chromosome rearrangements (translocations, inversions) Unable to detect low‐level mosaicism or tetraploidy
Karyotyping	Abnormalities detected throughout the entire genome Can detect low‐level mosaicism, tetraploidy Can detect Robertsonian translocations	Requires live cells, with high culture failure rate (up to 40%) Unable to detect microdeletion/duplications Unable to detect maternal cell contamination Time intensive (four weeks for results)

Couples who have an identified chromosomal rearrangement should be offered preimplantation genetic testing‐structural rearrangement (PGT‐SR) as a treatment option. PGT‐SR results in live birth rates ranging 31.7–52% following embryo transfer.^18^, ^19^, ^20^, ^21^

For RPL couples with a normal karyotype, PGT‐aneuploidy (PGT‐A) is promoted by some as a method of reducing pregnancy loss rates, by mediating the occurrence of aneuploidy (Table 6). In a recent retrospective analysis of PGT‐A in RPL patients by Murugappan et al,^22^ the incidence of not reaching a euploid transfer was 25% in women <35 years, and 37% in women ≥35 years, demonstrating the effect of age on oocyte quality and aneuploidy. When compared to expectant management, an intention‐to‐treat (ITT) analysis demonstrated no difference between groups for live birth or miscarriage rates. However, of the couples who completed PGT‐A and underwent a euploid transfer, the live birth rate was 57%; significantly higher compared to 34% in the control group.

**Table 6:** Summary of advantages and disadvantages of preimplantation genetic testing – aneuploidy (PGT‐A)

	Advantages	Disadvantages
PGT‐A	Used to evaluate ploidy status for all 23 chromosomes For patients reaching euploid transfer, may improve live birth rate Increased live birth rate in women >35 years Decrease in miscarriage rate in patients reaching euploid transfer Even if negative result, provides information to the patient	Invasive procedure with risks to patient and embryo No guarantee embryos will be suitable for biopsy or for transfer Risk of discarding usable embryos Cannot completely rule out aneuploidy due to challenge identifying mosaicism Not all platforms are equal, various techniques have different diagnostic strengths/weaknesses Cannot screen for single gene defect More costly than expectant management

Recommendations pertaining to chromosomal factors in RPL are listed in Table 7.

**Table 7:** Recommendations pertaining to chromosomal factors in recurrent pregnancy loss

Statement	Level of evidence
	Grade of consensus
Parental genetic abnormalities may be implicated in the aetiology of recurrent pregnancy loss	Level III evidence Consensus grade α
A detailed family history including consanguinity noting subfertility, stillbirths, miscarriages, neonatal deaths, disability or congenital anomalies is indicated in couples with recurrent pregnancy loss	GPP Consensus grade α
Karyotyping of both partners is indicated in couples with recurrent pregnancy loss	GPP Consensus grade α
Chromosomal assessment (via an array‐based technology) of products of conception should be offered in a subsequent pregnancy loss	GPP Consensus grade α
When a parental karyotypic abnormality is identified genetic counselling should be offered	GPP Consensus grade α
When parental karyotypic abnormality is identified preimplantation genetic testing (PGT) should be offered	GPP Consensus grade α
A discussion detailing the relative merits of PGT as a means to increase the probability of a successful pregnancy may be of value	Level II evidence Consensus grade β

### Anatomical factors

Anatomical abnormalities of the uterus and/or cervix, congenital or acquired, are associated with RPL (Table 8). Some of the fertility and obstetric implications of Müllerian anomalies are given in Table 9.

#### Congenital Müllerian anomalies

**Figure 1:**
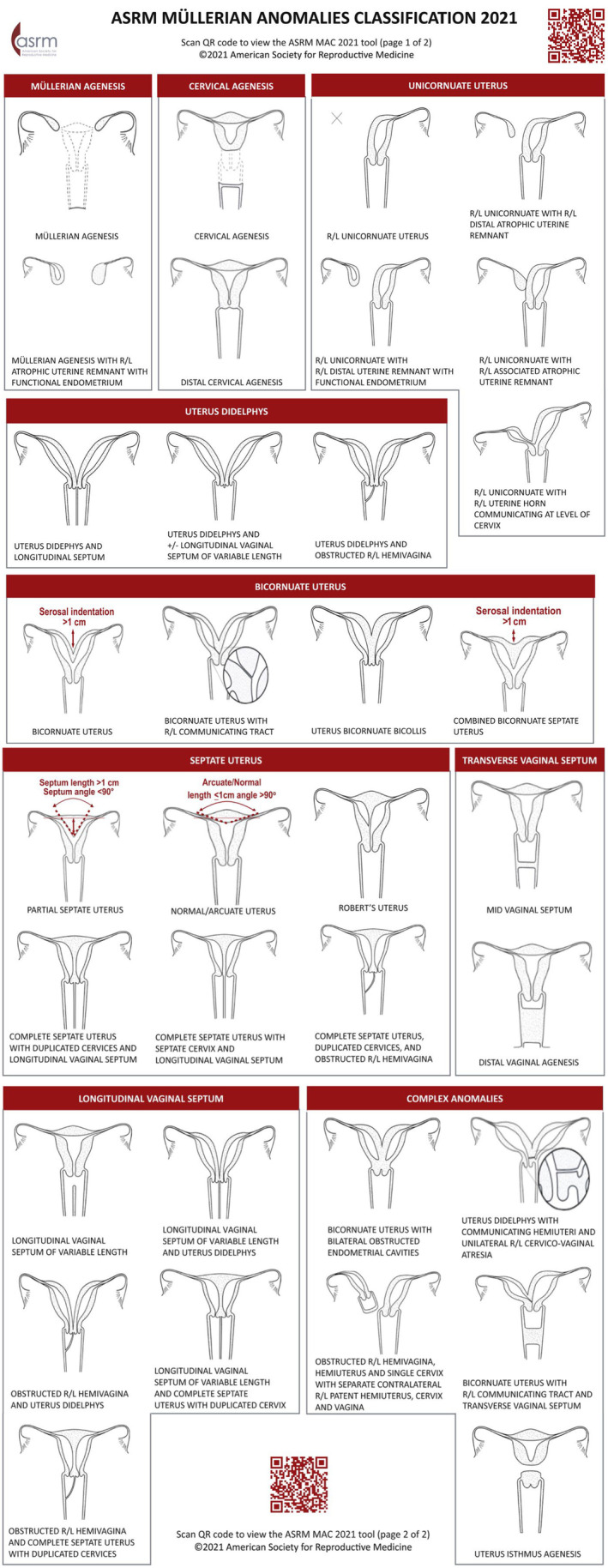
The American Fertility Society (ASRM) Müllerian Anomalies Classification 2021.

Embryologically, the uterus forms from two Müllerian ducts, which undergo formation and elongation, fusion and resorption. Failure of this process results in Müllerian anomalies, which are commonly classified according to the ASRM classification (Fig. 1).

Congenital uterine anomalies are present in 10–15% of the population of women who suffer from RPL, compared to approximately 7% of the general population.^23^, ^24^, ^25^ The exact mechanism for this is unclear, but may be related to impaired uterine distention, abnormal implantation, inflammation or decreased steroid hormone receptivity.^26^ The effect of reproductive tract anomalies on pregnancy outcomes have been largely derived from small observational studies.

#### Endometrial polyps

Endometrial polyps are the most common acquired uterine abnormality, affecting approximately 12% of the population.^27^, ^28^ Approximately 6–8.5% of women with RPL have uterine polyps,^29^, ^30^ suggesting a similar prevalence to the general population.

There is little data to suggest polyps are associated with increased risks of miscarriage or poor obstetric outcome. However, a randomised controlled trial (RCT) by Perez‐Medina (2005)^31^ observed a significantly higher pregnancy rate with intrauterine insemination following polypectomy, compared to polyp biopsy only in subfertile women (63% vs 28% respectively; *P* < 0.001).

#### Leiomyoma

Leiomyoma may affect pregnancy loss depending on type. As per the ACCEPT guidelines on fibroids in infertility, subserosal fibroids do not appear to affect fertility, intramural fibroids may possibly increase miscarriage rates, and submucosal fibroids significantly increase the risk of miscarriage.^32^ Proposed mechanisms included that they may act as an impediment to normal implantation due to position, may result in poor endometrial receptivity of the decidua overlying the myoma, and/or degeneration of the myoma leading to increased cytokine production.^33^

#### Intrauterine adhesions

Intrauterine adhesions have a prevalence of 1.5% as an incidental finding, but the prevalence is 21.5% for women who have had a postpartum curettage, and 4.1–12.5% for women with RPL.^34^ Intrauterine adhesions appear to cause infertility and RPL via abnormal implantation from denudation and poor vascularisation of the endometrium. Repeated curettage procedures have been identified as the biggest risk factor for intrauterine adhesion formation.^35^

**Table 8:** Recommendations pertaining to anatomical factors in recurrent pregnancy loss

Statement	Level of evidence
	Grade of consensus
Müllerian anomalies are associated with an increase in recurrent first trimester pregnancy loss	Level III‐2 Consensus grade β
Combined hysteroscopy and laparoscopy remains the gold standard for the investigation of Müllerian anomalies	GPP Consensus grade α
Sonohysterogram in combination with two‐dimensional ultrasound OR three‐dimensional ultrasound has a high sensitivity and specificity. As such, this would be an appropriate first‐line investigation	Level III‐3 Consensus grade α
Hysterosalpingogram is no longer a first‐line investigation as it gives no information about the fundal contour of the uterus, and also gives no information about other important aspects in fertility such as antral follicle count. It additionally exposes patients to radiation	GPP Consensus grade α
The evidence that resection of the uterine septum reduces recurrent pregnancy loss is uncertain	Level II Consensus grade α
However, the ACCEPT group believe that it is reasonable for hysteroscopic septum resection to be offered after appropriate counselling and discussion of risks and benefits	GPP Consensus grade α
Evidence for surgical correction of other uterine anomalies is poor	Level III‐3 Consensus grade α

**Table 9:** Fertility and obstetric implications of Müllerian anomalies

Classification	Distribution^96^	Fertility implications	Obstetric implications
Müllerian agenesis/cervical agenesis	4%	Associated with infertility, rather than recurrent pregnancy loss	
Unicornuate uterus	4.5%	Associated with first trimester loss (24%), second trimester loss (9%), and ectopic pregnancy (2%)^89^	Associated with preterm delivery (20%), and death *in utero* (10%)^89^ Higher rates of malpresentation^90^
Uterus didelphus	11%	Estimated miscarriage rate 32%^90^	Estimated preterm delivery rate of 28%^90^
Bicornuate uterus	46%	Estimated miscarriage rate 36%^90^	Estimated preterm delivery of 23%^90^
Septate uterus	37%	Partial septate uterus – most common type of anomaly, with poorest reproductive outcome.^90^ Estimated miscarriage rate up to 60% if untreated.^23^, ^91^, ^92^, ^93^, ^94^ Miscarriage classically occurs between 8–16 weeks^38^ Poorly understood mechanism, although thought to be due to poor implantation due to abnormal blood supply in septum and surrounding areas^38^, ^95^ Arcuate uterus – no association with first trimester pregnancy loss but may be associated with second trimester pregnancy loss^95^	Increased risk preterm delivery and malpresentation in partial septate uterus^95^

#### Investigations and diagnosis of anatomical anomalies^36^

Combined hysteroscopy and laparoscopy remain the gold standard for diagnosis of Müllerian anomalies.^37^, ^38^ However, two‐dimensional (2D)/three‐dimensional (3D) ultrasonography with sonohysterography has high sensitivity and specificity, and is therefore an appropriate non‐invasive investigation for cavity assessment of women with RPL.^37^ Other useful imaging modalities include sonohysterogram (especially in suspected fibroids) and magnetic resonance imaging (MRI) for further visualisation of the Müllerian duct abnormalities.

##### Management

###### Müllerian anomalies

The Randomised Uterine Septum Transsection (TRUST) trial did not support surgical correction of the uterine septum in a heterogenous cohort in order to decrease pregnancy loss and increase live births.^36^ However, there is supportive retrospective data. The evidence for the surgical correction of other Müllerian anomalies is not supportive.^25^, ^36^, ^38^, ^39^, ^40^, ^41^

Hysteroscopic approach is preferred for surgical management with overall low intraoperative and postoperative complications.^37^, ^38^, ^42^ The choice of energy source for resection should be that which is most familiar to the surgeon.^37^

###### Polyps

A Cochrane review recently determined that although there is no data on live birth rates following polypectomy, removal of an endometrial polyp may improve clinical pregnancy rates.^27^

###### Leiomyoma

The ACCEPT guidelines on fibroids in infertility acknowledge the requirement for individualised management of a women with a fibroid uterus.^32^ There is insufficient evidence for surgical management of intramural fibroids to be used as first‐line treatment to improve fertility. Hysteroscopic myomectomy of submucosal fibroids appears to improve pregnancy outcomes.^32^ Medical management is not recommended when prompt fertility is desired.^32^

###### Intrauterine adhesions

Hysteroscopic lysis of adhesions is recommended in cases of intrauterine adhesions.^43^ Although there are no RCTs for the management of intrauterine adhesions, several small studies report benefit to removing the adhesions. Pregnancy rates for women with RPL following hysteroscopic resection of intrauterine adhesions have been reported as 61.5%, with live birth rates of 71–75%.^44^, ^45^

### Endocrine Factors

Maternal endocrine disorders such as thyroid disease, hyperprolactinaemia, polycystic ovarian syndrome, and glucose intolerance have been associated with RPL. The management of these conditions, in addition to progesterone as support therapy are discussed below. The association between thyroid disorder and pregnancy loss or RPL is outlined in Table 10. Recommendations are given in Table 11.

**Table 10:** Association between thyroid disorder and PL or RPL

Thyroid disorder	Association	Management
Overt hypothyroidism	↑ PL	Levothyroxine improves MC rate
Subclinical hypothyroidism	Possible ↑ PL	Unclear if L‐T4 improves MC rate Recommendation to test thyroid antibodies (specifically TPOAb) if TSH is ≥4.0 mIU/L, and commence L‐T4 Could consider L‐T4 if TSH is >2.5 mIU/L and TPOAb positive
Hyperthyroidism	↑ PL	Consider definitive treatment (surgery), prior to pregnancy If on low‐dose anti‐thyroid therapy (eg carbimazole or propylthiouracil), recommendation to stop at pregnancy confirmation (as teratogenic), and monitor TFTs every two weeks until thyroid function has normalised. Can switch to or continue on the lowest effective dose of PTU if biochemical control is required for moderate to severe hyperthyroidism in the first trimester
Antibody‐positive euthyroid	↑RPL	Unclear if L‐T4 improves MC rate Test TSH every four weeks till mid‐gestation and consider L‐T4 if TSH is ≥4.0 mIU/L

L‐T4, levothyroxine; MC, miscarriage; mIU/L, milli‐international units per litre; PL, pregnancy loss; PTU, propylthiouracil; RPL, recurrent pregnancy loss; TFT, thyroid function test; TPOAb, thyroid peroxidase antibody test; TSH, thyroid stimulating hormone.

#### Thyroid disorders

Maternal thyroid production in the first trimester is vital for fetal neurocognitive development.^46^ Thyroid dysfunction appears to be implicated in RPL. The American Thyroid Association (ATA) revised their guidelines for the diagnosis and management of thyroid disease during pregnancy in 2017.^47^ These guidelines have been endorsed by the Council of the Endocrine Society of Australia,^48^ but there is no specific reference to fertility populations. Guidelines stipulate the use of population‐based trimester‐specific reference ranges for thyroid stimulating hormone (TSH).^47^ An upper limit of 4.0 mIU/L for TSH range applied from week 7 to week 12 has been revised (previously 2.5 mIU/L), with levels above this triggering initiation of treatment with levothyroxine (L‐T4).^47^

##### Hypothyroidism

Overt hypothyroidism is defined as an elevated TSH level with a low free thyroxine (T4) level. It is present in approximately 0.5% of all pregnant women, with Hashimoto's thyroiditis the most common cause.^49^ Untreated hypothyroidism is associated with an approximate two‐fold increased risk of pregnancy loss^50^ and treatment with L‐T4 reduces this risk. Negro et al.^51^ described rates of spontaneous pregnancy loss almost twice as high in untreated hypothyroid compared to euthyroid women, within a thyroid antibody‐negative cohort (6.1% vs 3.6% respectively; *P* = 0.006).

##### Subclinical hypothyroidism

Subclinical hypothyroidism (SCH) is defined as an elevated TSH with a normal free T4 level. Depending on clinical TSH reference ranges used, the prevalence of subclinical hypothyroidism is estimated to be 1.5–4% of the general pregnant population.^52^ This rate has been found to be considerably higher within the RPL population, affecting approximately 19% of this population in a cohort study.^53^ However, the combination of variance in the TSH range upper limit, in addition to differentiating the effect of SCH alone or with thyroid autoimmunity, has made the potential association with RPL unclear.

##### Hyperthyroidism

Overt hyperthyroidism is defined as a low TSH level with an elevated free T4 and/or free T3. It is present in 0.1–0.4% of pregnant women, with Graves' disease accounting for 85% of cases.^54^ Subclinical hyperthyroidism is defined as a low TSH with normal free thyroid hormone levels. Untreated or undertreated overt hyperthyroidism carries an increased risk of miscarriage.^55^ Subclinical hyperthyroidism is not associated with any adverse feto‐maternal outcomes.

##### Thyroid autoimmwunity (TAI)

Autoimmune thyroid disease occurs in 5–20% of women of child‐bearing age.^56^ Prevalence of TAI has been described as high as 17–33% within a RPL population.^57^ A large systematic review of 12 126 euthyroid women reported a strong association between maternal TAI and rates of pregnancy loss (odds ratio (OR) 3.9; 95% confidence interval (CI) 2.48–6.12; *P* < 0.001).^57^ This association was further increased for women with RPL (OR 4.22; CI 0.97–18.44; *P* = 0.006).

**Table 11:** Recommendations pertaining to thyroid factors in recurrent pregnancy loss (RPL)

Statement	Level of evidence
	Grade of consensus
In women with RPL, thyroid function tests (TSH and FT4) and thyroid antibodies (TPO and Tg) should be measured In the absence of population‐based reference ranges, a TSH ≥4.0 mIU/L should be considered abnormal If TSH is low/suppressed, then FT3 and TRAb should also be performed	GPP Consensus grade α
There is strong evidence that overt hypothyroidism or overt hyperthyroidism is associated with (R)PL Women with RPL with overt hypothryoidism or overt hyperthyroidism should be investigated and treated according to accepted guidelines^47^	Level I to III‐3 Consensus grade α
There is weak evidence that subclinical hypothyroidism (TSH ≥4.0 mIU/L; normal FT4/3; regardless of antibody status) is associated with RPL While the evidence for treatment with levothyroxine is weak, treatment in women with a TSH ≥4.0 mIU/L with an aim of reducing TSH to euthyroid levels is low risk and may reduce the risk of further loss	Level III‐3 Consensus grade α
There is weak evidence that a euthyroid but antibody‐positive state is associated with RPL. Two management strategies are suggested: a. Monitor TFTs every four weeks during pregnancy until mid‐gestation, and treat with levothyroxine if TSH ≥4.0 mIU/L b. Commence low‐dose levothyroxine (25–50 μg PO in the morning) with an aim of maintaining TSH <4.0 mIU/L	Level III‐3 Consensus grade β

FT3/4, free triiodothyronine 3/4; mIU/L, milli‐international units per litre; PO, per oral/orally; TFT, thyroid function test; Tg, thyroglobulin; TPO, thyroid peroxidase; TRAb, thyrotropin receptor antibodies; TSH, thyroid stimulating hormone.

###### Management

####### Overt hypothyroidism

L‐T4 is the recommended treatment of choice for maternal overt hypothyroidism, assuming there is adequate iodine intake.^58^ This is supported by Level 1 evidence which suggests L‐T4 reduces the risk of miscarriage by 81% (relative risk (RR) 0.19; 95% CI 0.08–0.39).^59^

####### Subclinical hypothyroidism

The evidence is conflicting regarding treatment of SCH. As per the ATA guidelines, it is recommended to initiate L‐T4 once TSH is ≥4.0 mIU/L, aiming for a therapeutic TSH target of 0.1–2.5 mIU/L.^47^ Additionally, women who have a TSH ≥4.0 mIU/L, or above the trimester‐ and population‐specific reference range, are recommended to have TPO antibodies measured.^47^

####### Hyperthyroidism

Current recommendations include achieving a euthyroid state prior to pregnancy. This is best achieved with endocrinologist involvement. The ATA suggest ceasing anti‐thyroid medications (eg carbimazole, propylthiouracil (PTU)) at diagnosis of pregnancy, due to teratogenicity.^47^

####### Thyroid autoimmunity

Despite the recognised association between thyroid antibodies and miscarriage, the management of this condition in euthyroid women is unclear.^60^, ^61^, ^62^ The ATA recommends regular assessment of TSH levels every four weeks until mid‐gestation within this population.^47^

#### Prolactin

The association between hyperprolactinaemia and RPL is tenuous (Table 12).^4^, ^5^, ^63^, ^64^

##### Management

There is weak evidence to suggest that normalising hyperprolactinaemia with a dopamine agonist can improve outcomes in RPL.^65^

**Table 12:** Recommendations pertaining to prolactin factors in recurrent pregnancy loss (RPL)

Statement	Level of evidence
	Grade of consensus
It is unclear if hyperprolactinaemia is implicated in RPL	Level IV Consensus grade α
Women with RPL should have prolactin levels obtained when there is clinical suspicion of hyperprolactinaemia	Good practice principle (GPP) Consensus grade α
For women with hyperprolactinaemia, consultation with an endocrinologist should be considered	GPP Consensus grade α
There is WEAK evidence to suggest treating hyperprolactinaemia may improve live birth rate	Level II Consensus grade α

#### Polycystic ovarian syndrome (PCOS)

PCOS is the most common endocrine disorder in women, affecting 8–13% of women of reproductive age (Table 13).^66^ The updated Australian PCOS Guidelines endorse the Rotterdam diagnostic criteria for adults.^66^

**Table 13:** Recommendations pertaining to polycystic ovarian syndrome (PCOS) in recurrent pregnancy loss

Statement	Level of evidence
	Grade of consensus
PCOS may be associated with pregnancy loss	Level IV Consensus grade α
The management of PCOS needs to be individualised but may include non‐pharmacological measures (such as diet and lifestyle interventions) and pharmacological measures (such as metformin)	GPP Consensus grade α

PCOS has been associated with a possible increase in the rate of spontaneous pregnancy loss, with a reported range of 25–37% for women with the condition compared to 18–25% of controls.^67^ As a heterogeneous syndrome, it is often difficult to discern the contributing factor of the PCOS entity alone on pregnancy loss, from common confounding conditions such as obesity,^68^ insulin resistance,^69^, ^70^, ^71^ and elevated luteinising hormone (LH) concentrations.^72^

##### Management

It is understood that weight loss improves ovulation rates in the obese population, and reduces fasting insulin concentrations.^73^, ^74^ Therefore, in the absence of high‐quality evidence for weight loss on RPL in PCOS individuals, women should be supported regardless to achieve a healthy body mass index (BMI). There is some evidence to support the use of metformin as an insulin sensitiser, to reduce pregnancy loss in women with PCOS.^71^, ^75^, ^76^

#### Obesity

Maternal obesity is associated with an increased risk of RPL in addition to other poor obstetric outcomes (Table 14). The exact pathophysiology is complex and poorly understood.^77^, ^78^, ^79^

##### Management

Weight loss among obese women with RPL is not well‐studied, with conflicting evidence.^73^, ^79^, ^80^, ^81^ Therefore, despite strong evidence to describe the association between obesity and RPL, the evidence to quantify the effects of weight loss to reverse this association is lacking. Regardless, the numerous benefits regarding healthy weight for both general health and in preparation for a pregnancy, ensures that aiming for a healthy BMI should be a first‐line recommendation in overweight and obese women with RPL.

**Table 14:** Recommendations pertaining to obesity in recurrent pregnancy loss (RPL)

Statement	Level of evidence
	Grade of consensus
Obesity is associated with RPL	Level III‐2 Consensus grade α
Individuals should be encouraged to achieve and maintain a normal body mass index, through weight loss interventions such as diet and exercise	Good practice principle Consensus grade α
There is some (weak) evidence to suggest weight loss can improve live birth rates	Level I Consensus grade α

#### Glucose intolerance

Glucose intolerance can present in several forms, including impaired fasting glucose, impaired glucose tolerance (IGT), and overt diabetes mellitus (DM). Several small observational studies have suggested a possible association between glucose intolerance and RPL, with prevalence observed between 11% to 27% in small case–controlled studies,^69^, ^82^, ^83^, ^84^ but some of this evidence is conflicting (Table 15).^85^

##### Management

Management should be coordinated by an endocrinologist to achieve a euglycaemic state, which may include metformin.^71^

**Table 15:** Recommendations pertaining to glucose intolerance in recurrent pregnancy loss (RPL)

Statement	Level of evidence
	Grade of consensus
There is conflicting evidence regarding whether glucose intolerance is associated with RPL	Level III‐2 Consensus grade α
Glucose intolerance should be screened with a fBGL and glycated haemoglobin (HbA1c). Consider a formal 75 g oGTT if either parameter is abnormal. If the 75 g oGTT is abnormal, referral to an endocrinologist is indicated	GPP Consensus grade α
Glucose impairment should be managed for general health benefits, and may also improve live birth rates in the context of RPL	GPP Consensus grade α

fBGL, fasting blood glucose level; GDM, gestational diabetes; HbA1c, glycated haemoglobin; oGTT, oral glucose tolerance test.

#### Progesterone

Progesterone is an essential hormone for normal pregnancy development. It has been hypothesised that administering exogenous progestogens, may support women with RPL by inducing secretory changes to the endometrium for successful implantation (Table 16).^86^

##### Management

A Cochrane literature review by Haas et al.^86^ concluded that progestogen supplementation may improve the rate of pregnancy loss in women with RPL compared with placebo; however, results did not reach statistical significance (20.1% vs 27.5%; RR 0.73; 95% CI 0.54–1.00; *P* = 0.10).

A study by Coomarasamy et al^87^ demonstrated a favourable impact on women with a threatened pregnancy loss who had had three or more previous pregnancy losses compared with placebo (71.5% vs 57.4%; RR 1.28; 95% CI 1.08–1.51), but not for women with 1–2 previous pregnancy losses.

**Table 16:** Recommendations pertaining to progesterone in in recurrent pregnancy loss (RPL)

Statement	Level of evidence
	Grade of consensus
The association between progesterone and RPL is uncertain	Level I Consensus grade α
Progesterone supplementation should be individualised If supplementing progesterone, progesterone and not progestins should be used The exact dose is uncertain; however, based on the PROMISE and PRISM trials, 400 mg twice daily per vagina is recommended Supplementation is recommended in women with ≥2 pregnancy losses especially in the setting of a threatened miscarriage	Level II Consensus grade α

## Supporting information


Appendix S1.

## References

[1] Royal College of Obstetricians & Gynaecologists (RCOG) . The Investigation and Treatment of Couples with Recurrent First Trimester and Second Trimester Miscarriage, Green‐top guideline no. 17 . 2011 [Accessed 20 June 2023]. Available from URL: https://ranzcog.edu.au/wp‐content/uploads/2022/05/The‐Investigation‐and‐Treatment‐of‐Couples‐with‐Recurrent‐First‐trimester‐and‐Second‐trimester‐Miscarriage.pdf.

[2] Little AB . There's many a slip 'twixt implantation and the crib. N Engl J Med 1988; 319(4): 241–242. 3393175 10.1056/NEJM198807283190409

[3] Stirrat GM . Recurrent miscarriage. Lancet 1990; 336(8716): 673–675. 1975862 10.1016/0140-6736(90)92159-f

[4] Practice Committee of the American Society for Reproductive M . Evaluation and treatment of recurrent pregnancy loss: A committee opinion. Fertil Steril 2012; 98(5): 1103–1111. 22835448 10.1016/j.fertnstert.2012.06.048

[5] European Society of Human Reproduction & Embryology (ESHRE) . Guideline on the management of recurrent pregnancy loss . 2022 [Accessed 20 June 2023]. Available from URL: https://www.eshre.eu/Guidelines‐and‐Legal/Guidelines/Recurrent‐pregnancy‐loss.

[6] van Dijk MM , Kolte AM , Limpens J *et al*. Recurrent pregnancy loss: Diagnostic workup after two or three pregnancy losses? A systematic review of the literature and meta‐analysis. Hum Reprod Update 2020; 26(3): 356–367. 32103270 10.1093/humupd/dmz048 PMC7161667

[7] Shekelle PG , Maglione MA , Luoto J . Global Health Evidence Evaluation Framework. Rockville (MD): Agency for Healthcare Research and Quality (US), 2013; Table B.9, NHMRC Evidence Hierarchy: designations of ‘levels of evidence’ according to type of research question (including explanatory notes) [Accessed 22 June 2023]. Available from URL: https://www.ncbi.nlm.nih.gov/books/NBK121300/table/appb.t21/. 23427349

[8] Philipp T , Philipp K , Reiner A *et al*. Embryoscopic and cytogenetic analysis of 233 missed abortions: Factors involved in the pathogenesis of developmental defects of early failed pregnancies. Hum Reprod 2003; 18(8): 1724–1732. 12871891 10.1093/humrep/deg309

[9] Sugiura‐Ogasawara M , Ozaki Y , Katano K *et al*. Abnormal embryonic karyotype is the most frequent cause of recurrent miscarriage. Hum Reprod 2012; 27(8): 2297–2303. 22661547 10.1093/humrep/des179

[10] Tur‐Torres MH , Garrido‐Gimenez C , Alijotas‐Reig J . Genetics of recurrent miscarriage and fetal loss. Best Pract Res Clin Obstet Gynaecol 2017; 42: 11–25. 28412101 10.1016/j.bpobgyn.2017.03.007

[11] Carp H , Feldman B , Oelsner G , Schiff E . Parental karyotype and subsequent live births in recurrent miscarriage. Fertil Steril 2004; 81(5): 1296–1301. 15136093 10.1016/j.fertnstert.2003.09.059

[12] Popescu F , Jaslow CR , Kutteh WH . Recurrent pregnancy loss evaluation combined with 24‐chromosome microarray of miscarriage tissue provides a probable or definite cause of pregnancy loss in over 90% of patients. Hum Reprod 2018; 33(4): 579–587. 29538673 10.1093/humrep/dey021

[13] Franasiak JM , Forman EJ , Hong KH *et al*. The nature of aneuploidy with increasing age of the female partner: A review of 15,169 consecutive trophectoderm biopsies evaluated with comprehensive chromosomal screening. Fertil Steril 2014; 101(3): 656–663.e1. 24355045 10.1016/j.fertnstert.2013.11.004

[14] Sugiura‐Ogasawara M , Ozaki Y , Sato T *et al*. Poor prognosis of recurrent aborters with either maternal or paternal reciprocal translocations. Fertil Steril 2004; 81(2): 367–373. 14967375 10.1016/j.fertnstert.2003.07.014

[15] Kabessa M , Harlev A , Friger M *et al*. Pregnancy outcomes among patients with recurrent pregnancy loss and chromosomal aberration (CA) without PGD. J Perinat Med 2018; 46(7): 764–770. 28672755 10.1515/jpm-2016-0408

[16] Franssen MT , Korevaar JC , Leschot NJ *et al*. Selective chromosome analysis in couples with two or more miscarriages: Case‐control study. BMJ 2005; 331(7509): 137–141. 15985440 10.1136/bmj.38498.669595.8F PMC558698

[17] Gardner M , Sutherland G , Shaffer L . Chromosome Abnormalities and Genetic Counselling, 4th edn. Oxford, UK: Oxford University Press, 2012.

[18] Chow JFC , Yeung WSB , Lee VCY *et al*. Evaluation of preimplantation genetic testing for chromosomal structural rearrangement by a commonly used next generation sequencing workflow. Eur J Obstet Gynecol Reprod Biol 2018; 224: 66–73. 29547808 10.1016/j.ejogrb.2018.03.013

[19] Christodoulou C , Dheedene A , Heindryckx B *et al*. Preimplantation genetic diagnosis for chromosomal rearrangements with the use of array comparative genomic hybridization at the blastocyst stage. Fertil Steril 2017; 107(1): 212–219.e3. 27793373 10.1016/j.fertnstert.2016.09.045

[20] Idowu D , Merrion K , Wemmer N *et al*. Pregnancy outcomes following 24‐chromosome preimplantation genetic diagnosis in couples with balanced reciprocal or Robertsonian translocations. Fertil Steril 2015; 103(4): 1037–1042. 25712573 10.1016/j.fertnstert.2014.12.118

[21] Li G , Jin H , Xin Z *et al*. Increased IVF pregnancy rates after microarray preimplantation genetic diagnosis due to parental translocations. Syst Biol Reprod Med 2014; 60(2): 119–124. 24377704 10.3109/19396368.2013.875241

[22] Murugappan G , Shahine LK , Perfetto CO *et al*. Intent to treat analysis of in vitro fertilization and preimplantation genetic screening versus expectant management in patients with recurrent pregnancy loss. Hum Reprod 2016; 31(8): 1668–1674. 27278003 10.1093/humrep/dew135

[23] Harger JH , Archer DF . Etiology of recurrent pregnancy losses and outcomes of subsequent pregnancies. Obstet Gynecol 1983; 62(5): 574–581. 6604890

[24] Acien PAM , Sanchez‐Ferrer M . Complex malformations of the femal genital tract. New types and revision of classification. Hum Reprod 2004; 19(10): 2377–2384. 15333604 10.1093/humrep/deh423

[25] Grimbizis GF , Camus M , Tarlatzis BC *et al*. Clinical implications of uterine malformations and hysteroscopic treatment results. Hum Reprod Update 2001; 7(2): 161–174. 11284660 10.1093/humupd/7.2.161

[26] Devi Wold AS , Pham N , Arici A . Anatomic factgors in recurrent pregnancy loss. Semin Reprod Med 2006; 24(1): 25–32. 16418975 10.1055/s-2006-931798

[27] Bosteels J , van Wessel S , Weyers S *et al*. Hysteroscopy for treating subfertility associated with suspected major uterine cavity abnormalities. Cochrane Database Syst Rev 2018; 12(12): CD009461. 30521679 10.1002/14651858.CD009461.pub4 PMC6517267

[28] Salim S , Won H , Nesbitt‐Hawes E *et al*. Diagnosis and management of endometrial polyps: A critical review of the literature. J Minim Invasive Gynecol 2011; 18(5): 569–581. 21783430 10.1016/j.jmig.2011.05.018

[29] Elsokkary M , Elshourbagy M , Labib K *et al*. Assessment of hysteroscopic role in management of women with recurrent pregnancy loss. J Matern Fetal Neonatal Med 2018; 31(11): 1494–1504. 28412850 10.1080/14767058.2017.1319925

[30] Seckin B , Sarikaya E , Oruc AS *et al*. Office hysteroscopic findings in patients with two, three, and four or more, consecutive miscarriages. Eur J Contracept Reprod Health Care 2012; 17(5): 393–398. 22974433 10.3109/13625187.2012.698767

[31] Perez‐Medina T , Bajo‐Arenas J , Salazar F *et al*. Endometrial polyps and their implication in the pregnancy rates of patients undergoing intrauterine insemination: A prospective, randomized study. Hum Reprod 2005; 20(6): 1632–1635. 15760959 10.1093/humrep/deh822

[32] Kroon B , Johnson N , Chapman M *et al*. Fibroids in infertility—consensus statement from ACCEPT (Australasian CREI Consensus Expert Panel on Trial evidence). Aust N Z J Obstet Gynaecol 2011; 51(4): 289–295. 21806566 10.1111/j.1479-828X.2011.01300.x

[33] Simpson JL . Causes of fetal wastage. Clin Obstet Gynecol 2007; 50(1): 10–30. 17304022 10.1097/GRF.0b013e31802f11f6

[34] Deans R , Abbott J . Review of intrauterine adhesions. J Minim Invasive Gynecol 2010; 17(5): 555–569. 20656564 10.1016/j.jmig.2010.04.016

[35] Hooker AB , Lemmers M , Thurkow AL *et al*. Systematic review and meta‐analysis of intrauterine adhesions after miscarriage: Prevalence, risk factors and long‐term reproductive outcome. Hum Reprod Update 2014; 20(2): 262–278. 24082042 10.1093/humupd/dmt045

[36] Rikken JFW , Kowalik CR , Emanuel MH *et al*. The randomised uterine septum transsection trial (TRUST): Design and protocol. BMC Womens Health 2018; 18(1): 163. 30290803 10.1186/s12905-018-0637-6 PMC6173848

[37] Budden A , Abbott JA . The diagnosis and surgical approach of uterine septa. J Minim Invasive Gynecol 2018; 25(2): 209–217. 28755995 10.1016/j.jmig.2017.07.017

[38] Homer HA , Li TC , Cooke ID . The septate uterus: A review of management and reproductive outcome. Fertil Steril 2000; 73(1): 1–14. 10632403 10.1016/s0015-0282(99)00480-x

[39] Acien P , Acien M . The presentation and management of complex female genital malformations. Hum Reprod Update 2016; 22(1): 48–69. 26537987 10.1093/humupd/dmv048

[40] Bhagavath B , Ellie G , Griffiths KM *et al*. Uterine malformations: An update of diagnosis, management, and outcomes. Obstet Gynecol Surv 2017; 72(6): 377–392. 28661551 10.1097/OGX.0000000000000444

[41] Hur C , Rehmer J , Flyckt R , Falcone T . Uterine factor infertility: A clinical review. Clin Obstet Gynecol 2019; 62(2): 257–270. 31021928 10.1097/GRF.0000000000000448

[42] Nouri K , Ott J , Huber JC *et al*. Reproductive outcome after hysteroscopic septoplasty in patients with septate uterus – a retrospective cohort study and systematic review of the literature. Reprod Biol Endocrinol 2010; 8: 52. 20492650 10.1186/1477-7827-8-52 PMC2885403

[43] Bailey AP , Jaslow CR , Kutteh WH . Minimally invasive surgical options for congenital and acquired uterine factors associated with recurrent pregnancy loss. Womens Health (Lond) 2015; 11(2): 161–167. 25776290 10.2217/whe.14.81

[44] Pabuccu R , Atay V , Orhon E *et al*. Hysteroscopic treatment of intrauterine adhesions is safe and effective in the restoration of normal menstruation and fertility. Fertil Steril 1997; 68(6): 1141–1143. 9418714 10.1016/s0015-0282(97)00375-0

[45] Yu D , Li TC , Xia E *et al*. Factors affecting reproductive outcome of hysteroscopic adhesiolysis for Asherman's syndrome. Fertil Steril 2008; 89(3): 715–722. https://pubmed.ncbi.nlm.nih.gov/17681324. 17681324 10.1016/j.fertnstert.2007.03.070

[46] Krog MC , Nielsen HS , Christiansen OB , Kolte AM . Reproductive endocrinology in recurrent pregnancy loss. Clin Obstet Gynecol 2016; 59(3): 474–486. 27403585 10.1097/GRF.0000000000000225

[47] Alexander EK , Pearce EN , Brent GA *et al*. 2017 guidelines of the American Thyroid Association for the diagnosis and Management of Thyroid Disease during Pregnancy and the postpartum. Thyroid 2017; 27(3): 315–389. 28056690 10.1089/thy.2016.0457

[48] Hamblin PS , Sheehan PM , Allan C *et al*. Subclinical hypothyroidism during pregnancy: The Melbourne public hospitals consensus. Intern Med J 2019; 49(8): 994–1000. 30561039 10.1111/imj.14210

[49] Stagnaro‐Green A , Pearce E . Thyroid disorders in pregnancy. Nat Rev Endocrinol 2012; 8(11): 650–658. 23007317 10.1038/nrendo.2012.171

[50] Stagnaro‐Green A . Thyroid antibodies and miscarriage: Where are we at a generation later? J Thyroid Res 2011; 2011: 841949. 21687610 10.4061/2011/841949 PMC3112530

[51] Negro R , Schwartz A , Gismondi R *et al*. Increased pregnancy loss rate in thyroid antibody negative women with TSH levels between 2.5 and 5.0 in the first trimester of pregnancy. J Clin Endocrinol Metab 2010; 95(9): E44–E48. 20534758 10.1210/jc.2010-0340

[52] Negro R , Mestman JH . Thyroid disease in pregnancy. Best Pract Res Clin Endocrinol Metab 2011; 25(6): 927–943. 22115167 10.1016/j.beem.2011.07.010

[53] Bernardi LA , Cohen RN , Stephenson MD . Impact of subclinical hypothyroidism in women with recurrent early pregnancy loss. Fertil Steril 2013; 100(5): 1326–1331. 23954357 10.1016/j.fertnstert.2013.07.1975

[54] Lazarus JH . Thyroid function in pregnancy. Br Med Bull 2011; 97: 137–148. 21186204 10.1093/bmb/ldq039

[55] Negro R , Stagnaro‐Green A . Clinical aspects of hyperthyroidism, hypothyroidism, and thyroid screening in pregnancy. Endocr Pract 2014; 20(6): 597–607. 24449669 10.4158/EP13350.RA

[56] Akhtar MA , Agrawal R , Brown J *et al*. Thyroxine replacement for subfertile women with euthyroid autoimmune thyroid disease or subclinical hypothyroidism. Cochrane Database Syst Rev 2019; 6(6): CD011009. 31236916 10.1002/14651858.CD011009.pub2 PMC6591496

[57] Thangaratinam S , Tan A , Knox E *et al*. Association between thyroid autoantibodies and miscarriage and preterm birth: Meta‐analysis of evidence. BMJ 2011; 342: d2616. 21558126 10.1136/bmj.d2616 PMC3089879

[58] De Groot L , Abalovich M , Alexander EK *et al*. Management of thyroid dysfunction during pregnancy and postpartum: An Endocrine Society clinical practice guideline. J Clin Endocrinol Metab 2012; 97(8): 2543–2565. 22869843 10.1210/jc.2011-2803

[59] Vissenberg R , van den Boogaard E , van Wely M *et al*. Treatment of thyroid disorders before conception and in early pregnancy: A systematic review. Hum Reprod Update 2012; 18(4): 360–373. 22431565 10.1093/humupd/dms007

[60] Dizon‐Townson DMC , Sibai B , Spong CY *et al*. The relationship between the factor V leiden mutation and pregnancy outcomes for mother and fetus. Obstet Gynecol 2005; 106(3): 517–524. 16135581 10.1097/01.AOG.0000173986.32528.ca

[61] Rao M , Zeng Z , Zhou F *et al*. Effect of levothyroxine supplementation on pregnancy loss and preterm birth in women with subclinical hypothyroidism and thyroid autoimmunity: A systematic review and meta‐analysis. Hum Reprod Update 2019; 25(3): 344–361. 30951172 10.1093/humupd/dmz003

[62] Wang H , Gao H , Chi H *et al*. Effect of levothyroxine on miscarriage among women with Normal thyroid function and thyroid autoimmunity undergoing in vitro fertilization and embryo transfer: A randomized clinical trial. JAMA 2017; 318(22): 2190–2198. 29234808 10.1001/jama.2017.18249

[63] Bussen S , Sutterlin M , Steck T . Endocrine abnormalities during the follicular phase in women with recurrent spontaneous abortion. Hum Reprod 1999; 14(1): 18–20. 10374087 10.1093/humrep/14.1.18

[64] Li W , Ma N , Laird SM *et al*. The relationship between serum prolactin concentration and pregnancy outcome in women with unexplained recurrent miscarriage. J Obstet Gynaecol 2013; 33(3): 285–288. 23550860 10.3109/01443615.2012.759916

[65] Hirahara F , Andoh N , Sawai K *et al*. Hyperprolactinemic recurrent miscarriage and results of randomized bromocriptine treatment trials. Fertil Steril 1998; 70(2): 246–252. 9696215 10.1016/s0015-0282(98)00164-2

[66] Teede HJ , Misso ML , Costello MF *et al*. Recommendations from the international evidence‐based guideline for the assessment and management of polycystic ovary syndrome. Fertil Steril 2018; 110(3): 364–379. 30033227 10.1016/j.fertnstert.2018.05.004 PMC6939856

[67] Homburg R . Pregnancy complications in PCOS. Best Pract Res Clin Endocrinol Metab 2006; 20(2): 281–292. 16772158 10.1016/j.beem.2006.03.009

[68] Wang JX , Davies MJ , Norman RJ . Polycystic ovarian syndrome and the risk of spontaneous abortion following assisted reproductive technology treatment. Hum Reprod 2001; 16(12): 2606–2609. 11726582 10.1093/humrep/16.12.2606

[69] Craig LB , Ke RW , Kutteh WH . Increased prevalence of insulin resistance in women with a history of recurrent pregnancy loss. Fertil Steril 2002; 78(3): 487–490. 12215322 10.1016/s0015-0282(02)03247-8

[70] Wani AA , Gul I , Jabeen F *et al*. Relationship of insulin resistance with recurrent pregnancy loss. Int J Reprod Contracept Obstet Gynecol 2017; 6(4): 1312.

[71] Zolghadri J , Tavana Z , Kazerooni T *et al*. Relationship between abnormal glucose tolerance test and history of previous recurrent miscarriages, and beneficial effect of metformin in these patients: A prospective clinical study. Fertil Steril 2008; 90(3): 727–730. 18001723 10.1016/j.fertnstert.2007.06.079

[72] Regan L , Owen EJ , Jacobs HS . Hypersecretion of luteinising hormone, infertility, and miscarriage. Lancet 1990; 336(8724): 1141–1144. 1978024 10.1016/0140-6736(90)92765-a

[73] Best D , Avenell A , Bhattacharya S . How effective are weight‐loss interventions for improving fertility in women and men who are overweight or obese? A systematic review and meta‐analysis of the evidence. Hum Reprod Update 2017; 23(6): 681–705. 28961722 10.1093/humupd/dmx027

[74] Clark AM , Ledger W , Galletly C *et al*. Weight loss results in significant improvement in pregnancy and ovulation rates in anovulatory obese women. Hum Reprod 1995; 10(10): 2705–2712. 8567797 10.1093/oxfordjournals.humrep.a135772

[75] Glueck CJ , Phillips H , Cameron D *et al*. Continuing metformin throughout pregnancy in women with polycystic ovary syndrome appears to safely reduce first‐trimester spontaneous abortion: A pilot study. Fertil Steril 2001; 75(1): 46–52. 11163815 10.1016/s0015-0282(00)01666-6

[76] Nawaz FH , Khalid R , Naru T , Rizvi J . Does continuous use of metformin throughout pregnancy improve pregnancy outcomes in women with polycystic ovarian syndrome? J Obstet Gynaecol Res 2008; 34(5): 832–837. 18834342 10.1111/j.1447-0756.2008.00856.x

[77] Boots CE , Bernardi LA , Stephenson MD . Frequency of euploid miscarriage is increased in obese women with recurrent early pregnancy loss. Fertil Steril 2014; 102(2): 455–459. 24907916 10.1016/j.fertnstert.2014.05.005

[78] Broughton DE , Moley KH . Obesity and female infertility: Potential mediators of obesity's impact. Fertil Steril 2017; 107(4): 840–847. 28292619 10.1016/j.fertnstert.2017.01.017

[79] Cavalcante MB , Sarno M , Peixoto AB *et al*. Obesity and recurrent miscarriage: A systematic review and meta‐analysis. J Obstet Gynaecol Res 2019; 45(1): 30–38. 30156037 10.1111/jog.13799

[80] Clark AM , Thornley B , Tomlinson L *et al*. Weight loss in obese infertile women results in improvement in reproductive outcome for all forms of fertility treatment. Hum Reprod 1998; 13(6): 1502–1505. 9688382 10.1093/humrep/13.6.1502

[81] Einarsson S , Bergh C , Friberg B *et al*. Weight reduction intervention for obese infertile women prior to IVF: A randomized controlled trial. Hum Reprod 2017; 32(8): 1621–1630. 28854592 10.1093/humrep/dex235

[82] Ispasoiu CA , Chicea R , Stamatian FV , Ispasoiu F . High fasting insulin levels and insulin resistance may be linked to idiopathic recurrent pregnancy loss: A case‐control study. Int J Endocrinol 2013; 2013: 576926. 24371440 10.1155/2013/576926 PMC3858974

[83] Matjila MJ , Hoffman A , van der Spuy ZM . Medical conditions associated with recurrent miscarriage‐is BMI the tip of the iceberg? Eur J Obstet Gynecol Reprod Biol 2017; 214: 91–96. 28494269 10.1016/j.ejogrb.2017.05.003

[84] Romero ST , Sharshiner R , Stoddard GJ *et al*. Correlation of serum fructosamine and recurrent pregnancy loss: Case‐control study. J Obstet Gynaecol Res 2016; 42(7): 763–768. 26935884 10.1111/jog.12974

[85] Wang Y , Zhao H , Li Y *et al*. Relationship between recurrent miscarriage and insulin resistance. Gynecol Obstet Investig 2011; 72(4): 245–251. 21952420 10.1159/000325165

[86] Haas DM , Hathaway TJ , Ramsey PS . Progestogen for preventing miscarriage in women with recurrent miscarriage of unclear etiology. Cochrane Database Syst Rev 2019; 2019(11): CD003511. 31745982 10.1002/14651858.CD003511.pub5 PMC6953238

[87] Coomarasamy A , Devall AJ , Cheed V *et al*. A randomized trial of progesterone in women with bleeding in early pregnancy. N Engl J Med 2019; 380(19): 1815–1824. 31067371 10.1056/NEJMoa1813730

[88] Hong Li Y , Marren A . Recurrent pregnancy loss: A summary of international evidence‐based guidelines and practice. Aust J Gen Pract 2018; 47(7): 432–436. 30114870 10.31128/AJGP-01-18-4459

[89] Reichman D , Laufer MR , Robinson BK . Pregnancy outcomes in unicornuate uteri: A review. Fertil Steril 2009; 91(5): 1886–1894. 18439594 10.1016/j.fertnstert.2008.02.163

[90] Fedele L , Zamberletti D , Vercellini P *et al*. Reproductive performance of women with unicornuate uterus. Fertil Steril 1987; 47(3): 416–419. 3556620 10.1016/s0015-0282(16)59047-5

[91] Golan A , Langer R , Bukovsky I , Caspi E . Congenital anomalies of the mullerian system. Fertil Steril 1989; 51(5): 747–755. 2651163 10.1016/s0015-0282(16)60660-x

[92] Green LK , Harris RE . Uterine anomalies. Frequency of diagnosis and associated obstetric complications. Obstet Gynecol 1976; 47(4): 427–429. 1256726

[93] Heinonen PK , Saarikoski S , Pystynen P . Reproductive performance of women with uterine anomalies. An evaluation of 182 cases. Acta Obstet Gynecol Scand 1982; 61(2): 157–162. 7113692 10.3109/00016348209156548

[94] Proctor JA , Haney AF . Recurrent first trimester pregnancy loss is associated with uterine septum but not with bicornuate uterus. Fertil Steril 2003; 80(5): 1212–1215. 14607577 10.1016/s0015-0282(03)01169-5

[95] Chan YY , Jayaprakasan K , Tan A *et al*. Reproductive outcomes in women with congenital uterine anomalies: A systematic review. Ultrasound Obstet Gynecol 2011; 38(4): 371–382. 21830244 10.1002/uog.10056

[96] Acien P . Incidence of Mullerian defects in fertile and infertile women. Hum Reprod 1997; 12(7): 1372–1376. 9262259 10.1093/oxfordjournals.humrep.a019588

