# Supplementary material for: Australasian Recurrent Pregnancy Loss Clinical Management Guideline 2024 Part I
Source: Aust N Z J Obstet Gynaecol. 2024 Jun 27;64(5):432–44. doi: 10.1111/ajo.13821 (PMC11660023; doi:10.1111/ajo.13821)
Supplement: Supplementary file 1 — Appendix S1. [file AJO-64-432-s001.docx]

Appendix

Search terms used to search MEDLINE, EMBASE, PubMed and the Cochrane Database of Systematic Reviews

| Aetiology | Search terms |
| --- | --- |
| Chromosomal | “chromosomal anomaly”, “chromosomal abnormality”, “genetic$”, “genetic anomaly”, “genetic abnormality”, “aneuploidy”, “preimplantation genetic diagnosis”, “preimplantation genetic screening”, “translocation$”, “preimplantation genetic diagnosis”, “PGD”, “preimplantation genetic testing”, “structural chromosom$” |
| Anatomy | “uterine anomal$”, “pregnancy”, “unicornuate uter$”, “mullerian duct anomal$”, “mullerian anomal$”,“unicornuate”, “bicornuate”, “didelphys”, “septate”, “hypoplasia”, “agenesis”, “arcuate”, “investigat*”, “diagnose”, “uterine malformation$”, “treatment”, “sept$ uter$”, “hysteroscop$ findings”, “polyp$”, “polypectomy”, “fertility”, “acquire uterine anomal$”, “fibroid$”, “leiomyoma”, “intrauterine adhesions”, “asherman$”, “uterine synechia” |
| Endocrine | “Thyroid”, “hypothyroid”, “pregnan$”, “subclinical hypothyroid$”, “miscarriage”, “pregnancy”, “treatment”, “recurrent pregnancy loss”, “recurrent miscarriage”, “management”, “prolactin”, “$prolactin$”,“miscarriage”, “bromocriptine”, “dopamine agonist”, ”polycystic ovar$”, “obesity”, “management”, “treatment”, “intervention”, “exercise”, “obes$”, “weight loss”, “lifestyle”, “ovarian drilling”, “glucose”, “insulin”, “diabetes”, “T2DM”, “diabetes mellitus”, “hyperinsulin$”, “hyperglyc$”, “impaired glucose tolerance”, “metformin” |
| Thrombophilia | “Factor V leiden”, “Factor V leiden mutation”, “Factor V leiden heterozygous”, “Prothrombin”, “prothrombin mutation”, “prothrombin gene”, “Factor II mutation”, “Protein C deficiency”, Protein S deficiency”, “protein C”, protein C”, “MTHFR”, “Methylenetetrahydrofolate Reductase”, “Antiphospholipid”, “Clexane”, “heparin”, “aspirin”, “antithrombotic”, “hereditary thrombophilia”, “congenital thrombophilia” |
| Autoimmune disorders | “immune”, “human leukocyte antigen”, “human leukocyte antibody”, “natural killer cells”, “killer immunoglobulin-like cells”, “cytokine$”, “donor cell”, “paternal cell”, “immuni$”, “Immunotherapy”, “immunomodulation”, “immunoglobulin”, “coeliac”, “celiac”, “Steroid”, “prednisone”, “prednisolone”, “corticosteroid$”, “lipid”, “intralipid. “Granulocyte colony-stimulating factor)” |
| Infective, inflammatory, and endometrial causes | “Infection”, “infective”, “infect$”, “microbiome”, “chronic endometritis”, “antibiotic$”, “Gardnerella vaginosis”, “cytomegalovirus”, “toxoplasmosis”, “toxoplasma”, “chlamydia”, “mycoplasma”, “ureaplasma”, “bacterial vaginosis”, “inflamm$”, “endometriosis”, “adenomyosis” |
| Environmental and lifestyle factors | “Environment”, “occupation$”, “pollution”, “bisphenol a”, “BPA”, “phthalate$”, “heavy metal$”, “metal”, “cadmium”, “lead”, “mercury”, “lifestyle”, “smok$”, “smoking”, “cigarette”, “tobacco”, “nicotine”, “caffeine”, “coffee”, “alcohol”, “stress”, “telomere”, “psychological”, “cortisol”, “tender loving care”, “TLC”, “support” |
| Male factor | “Sperm”, “semen”, “male factor”, “semen analysis”, “sperm morphology”, “DNA fragmentation”, “deoxyribonucleic acid fragmentation”, “sperm aneuploidy”, “sperm quality”, “obese$”, “BMI”, “smoking”, “cigarette”, “alcohol”, “weight loss”, “diet”, “antioxidants”, “varicocele” |
